# Setup and Analysis of Human Tonsil and Adenoid‐Based Immune Organoids for Studying T and B Cell‐Mediated Adaptive Immunity

**DOI:** 10.1002/cpz1.70485

**Published:** 2026-10-24

**Authors:** Maximilian Moll, Klaus W. Eichhorn, Thorsten Send, Dirk Baumjohann

**Affiliations:** ^1^ Medical Clinic III for Oncology, Hematology, Immuno‐Oncology and Rheumatology University Hospital Bonn, University of Bonn Bonn Germany; ^2^ Department of Otorhinolaryngology, Head and Neck Surgery University Hospital Bonn, University of Bonn Bonn Germany; ^3^ Department of Otorhinolaryngology/Head and Neck Surgery Johanniter‐Waldkrankenhaus Bonn Germany

**Keywords:** cell culture, GC, human immunology, immune organoid, Tfh cell

## Abstract

In recent years, demand for human‐based in vitro models has grown substantially. In this regard, human secondary lymphoid organ (SLO)‐derived ex vivo immune organoids are gaining recognition, as they provide a platform for studying highly differentiated, tissue‐specific immune cell subsets found in SLOs, such as lymph nodes, tonsils, or spleen. These systems support the study of adaptive immune responses mediated by T and B cells, e.g., due to the presence of T follicular helper cells and germinal center B cells within these immune organoids. Here, we describe procedures for the isolation of human tonsil‐ or adenoid‐derived immune cells, their cryopreservation and thawing, the establishment of immune organoids in vitro, and a detailed flow cytometry protocol for longitudinal phenotypic profiling of the immune organoids. In summary, these protocols serve as a foundation for downstream functional assays and complementary analyses in a human‐first approach. © 2026 The Author(s). *Current Protocols* published by Wiley Periodicals LLC.

**Basic Protocol 1**: Cell isolation from human tonsils and adenoids

**Basic Protocol 2**: Thawing of cryopreserved tonsil and adenoid cells and establishment of immune organoids

**Basic Protocol 3**: Flow cytometry for longitudinal phenotypical profiling of human tonsil/adenoid‐derived immune organoids.

## INTRODUCTION

Understanding the human immune system is the central aim of immunological research. To answer unresolved questions about our immune system, such as immune cell dynamics, infectious diseases, or autoimmunity, various model systems have been developed. One of the most studied and understood model systems is the mouse (Medetgul‐Ernar & Davis, 2022), which helped answer many important questions about the structure and dynamics of the immune system. Despite fundamental similarities between the mouse and human immune systems, key differences exist that oftentimes complicate the translation to human immunology. For example, class‐switching is fundamentally different, as both species have four IgG subclasses; however, these are not direct homologs (Martin & Lew, 1998). Furthermore, IL‐4 induces IgG1 and IgE in mice, but IgG4 and IgE in humans, which is relevant for allergy models (Mestas & Hughes, 2004).

Species‐specific differences also extend to the expression of key surface receptors and production of cytokines by T cells (Mestas & Hughes, 2004). CD28 is expressed on nearly all CD4^+^ and CD8^+^ T cells in mice, whereas in humans only ∼80% of CD4^+^ T cells and ∼50% of CD8^+^ T cells are CD28‐positive (Lenschow et al., 1996). This highlights the need for human‐based models that more faithfully recapitulate the human immune system. Human immunological research has long been confined to the analysis of peripheral blood, relying primarily on peripheral blood mononuclear cells (PBMCs) (Farber, 2021). However, circulating PBMCs are different from the highly differentiated, tissue‐specific immune cell populations found in secondary lymphoid organs (SLOs) such as lymph nodes, tonsils, or spleen, including germinal center (GC) B cells and T follicular helper (Tfh) cells (Farber, 2021; Victora & Nussenzweig, 2022). These tissue‐specific immune cells are central to the adaptive immune response as they drive the GC reaction within the B cell follicles of SLOs and give rise to class‐switched high‐affinity antibodies and long‐lived plasma cells (Baumjohann & Fazilleau, 2021).

In the 1990s, HIV researchers were among the first to exploit human palatine tonsil and adenoid (pharyngeal tonsil) tissue obtained from routine tonsillectomies and adenoidectomies for studying HIV pathogenesis (Glushakova et al., 1995). To this end, tissue was cut into small blocks and cultured on collagen sponges at an air‐liquid interface to mimic the natural physiology of the organ, an approach termed human lymphoid histoculture (HLH) (Glushakova et al., 1995). This system enabled the study of HIV infection dynamics within a tissue context. However, poor tissue perfusion resulted in limited viability and shortened culture duration (Schmidt et al., 2020). In the early 2000s, the system was advanced by dissociating tissue into single‐cell suspensions and culturing them for extended periods as human lymphoid aggregate culture (HLAC), substantially improving viability (Eckstein et al., 2001). As the demand for human‐based immune models grew, HLAC attracted renewed interest from immunologists ∼2020 to study adaptive immunity (Schmidt et al., 2020), with such cultures being increasingly referred to as “immune organoids” (Wagar et al., 2021). While immune organoids more closely resemble an assembloid rather than a canonical organoid that develops from stem cells under defined culturing conditions, they have enabled in‐depth investigation of tissue‐specific adaptive immunity by flow cytometry, bulk RNA‐sequencing, single‐cell RNA sequencing, and CRISPR‐Cas9‐mediated genetic manipulation of immune cell populations within the immune organoids (Bracey et al., 2026; Moll & Baumjohann, 2025; Morath et al., 2024; Schmidt & Baumjohann, 2022; Schmidt et al., 2020; Wagar et al., 2021).

Currently, few detailed protocols exist for immune cell isolation from tonsils and adenoids, immune organoid culture establishment, and systematic immunophenotyping of the resulting organoids with a focus on Tfh cell and GC biology. To fill this gap, we present three protocols: Basic Protocol 1 covers immune cell isolation from tonsils and adenoids, Basic Protocol 2 describes thawing of cryopreserved primary cells and immune organoid establishment, and Basic Protocol 3 provides a flow cytometry workflow for longitudinal phenotypic profiling of human tonsil‐ and adenoid‐derived immune organoids. These protocols are intended to support reproducible, uniformly designed studies with immune organoids.

*NOTE*: The study was approved by the local ethics committee of the Medical Faculty of the University of Bonn and prior informed consent was obtained from the children's legal guardians in written form. The study was conducted in accordance with the principles outlined in the Declaration of Helsinki.

*NOTE*: Appropriate informed consent is necessary for obtaining and use of human study material.

## Basic Protocol 1 CELL ISOLATION FROM HUMAN TONSILS AND ADENOIDS

This protocol is intended to describe the processing of human tonsil/adenoid tissue and isolation of immune cells for the establishment of human tonsil/adenoid‐based immune organoids. The multistep workflow is illustrated in Figure 1. This method allows for reproducible isolation of major immune cell subsets present in tonsils/adenoids.

**Figure 1:**
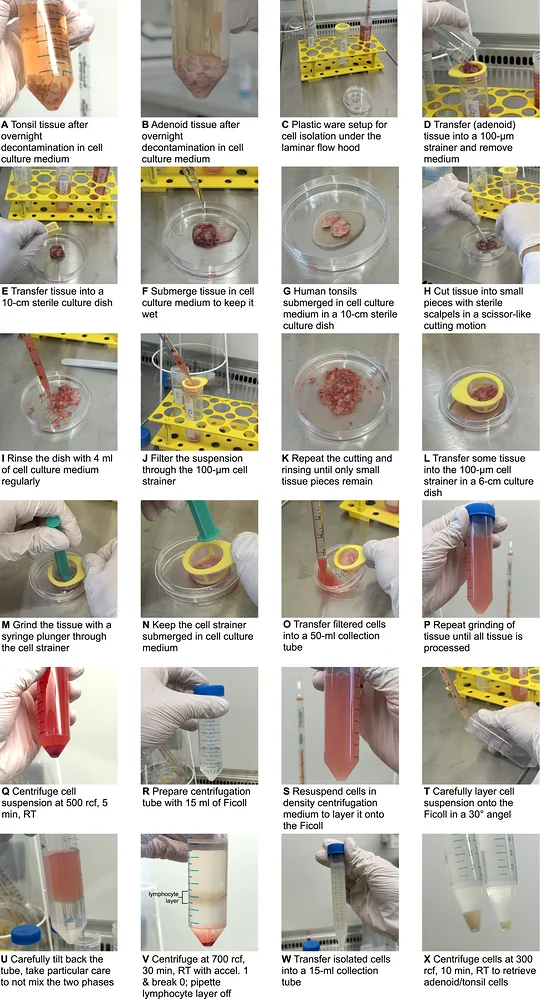
Cell isolation from human tonsils and adenoids. Description of the individual steps of Basic Protocol 1 for the isolation of tonsil/adenoid‐derived cells required for the setup of immune organoids.

### Materials

Fresh human tonsil or adenoid tissue
1× phosphate‐buffered saline (PBS) (Thermo Fisher Scientific, cat. no. 14190169)
Cell isolation medium (see recipe)
Penicillin‐streptomycin, 10,000 U/ml (Gibco, cat. no. 15140122)
Cytiva Ficoll‐Paque PLUS medium (Merck, cat. no. GE17‐1440‐02)
Density centrifugation medium (see recipe)
Trypan blue solution, 0.4% (Gibco, cat. no. 15250061)
Cryo medium (see recipe)

50‐ml centrifuge tubes (Greiner Bio‐One, cat. no. 227261)
Laminar flow hood
100‐µm cell strainer (VWR, cat. no. 732‐2759)
Refrigerator, 4°C
10‐cm cell culture dishes (Sarstedt, cat. no. 83.3902)
Sterile scalpels (Swann‐Morton, cat. no. 0507)
6‐cm cell culture dish (VWR, cat. no. 734‐2318)
Syringe plunger (Braun, cat. no. 12732637)
Centrifuge (Eppendorf, cat. no. 5811000015)
10‐ml serological pipette (Greiner Bio‐One, cat. no. 607180)
15‐ml centrifuge tubes (Greiner Bio‐One, cat. no. 188271)
Ice bucket with ice
96‐well V‐bottom plates (Sarstedt, cat. no. 82.1583)
20‐µl piston pipette (Mettler Toledo, cat. no. 17011788)
20‐µl pipette tips (Mettler Toledo, cat. no. 30389225)
Luna‐FX7 reusable cell counter slide (Logos Biosystems, cat. no. L12011)
Luna‐FX7 automated cell counter (Logos Biosystems)
Cryotubes (Sarstedt, cat. no. 72.380)
Cryotube racks (Sarstedt, cat. no. 93.856.040)
Cryotube labels
Cryotube color coding chips (Sarstedt, cat. no. 65.386.992)
Multipette M4 repeating pipette (Eppendorf, cat. no. 4982000012)
Corning CoolCell FTS30 freezing container (Corning, cat. no. CLS432008)
Freezer, –80°C
Liquid nitrogen storage or –150°C freezer

#### Tissue processing

1 Collect resected tonsil or adenoid specimen in sterile 50‐ml centrifuge tubes filled with 40 ml of chilled sterile PBS directly in the operating room. Store tissue at 4°C and proceed within the same day after surgery. Handle tonsil tissue in a laminar flow hood under sterile conditions for all further processing steps.
  If sterile tubes filled with PBS are not available in the operating room, samples should be transferred into PBS or medium as quickly as possible.
2 Remove the PBS by pouring the adenoid/tonsil tissue‐containing samples onto a 100‐µm cell strainer and discard the flow through.
3 Transfer the tissue into a sterile 50‐ml centrifuge tube and add 20 to 30 ml of cell isolation medium to the tube, depending on the size of the tissue. The tissue should be fully submerged in the medium for decontamination. For whole tonsils, increase the penicillin/streptomycin content from 1× to 10×. Incubate overnight at 4°C in the fridge (Fig. 1A‐B).
4 Prepare plasticware needed for isolation of tonsil/adenoid cells (Fig. 1C)
5 The next morning, remove medium used for decontamination using a 100‐µm cell strainer, discard medium, and collect the tissue in the cell strainer (Fig. 1D).
6 Transfer tissue into a sterile 10‐cm culture dish and add 2 ml of cell isolation medium onto tissue to keep it wet during processing (Fig. 1E‐G).
7 Prepare a fresh 50‐ml centrifuge tube to collect all cells. Place the used 100‐µm cell strainer onto the centrifuge tube for further straining of the cell solution.
8 Remove bloody capsular and cauterized parts of the tissue with sterile scalpels and discard them.
9 Cut tissue into small pieces with two sterile scalpels in a scissor‐like cutting motion (Fig. 1H) and regularly rinse the dish with 4 ml of complete medium (Fig. 1I). Filter the suspension through the 100‐µm cell strainer (Fig. 1J). Repeat the cutting and rinsing until only small tissue pieces remain (Fig. 1K). At the end, keep these tissue pieces submerged in 2 ml complete medium.
10 For further mechanical disruption of the tissue, transfer the 100‐µm cell strainer from the centrifuge tube into a new sterile 6‐cm cell culture dish. Transfer some of the tissue pieces with the scalpels into the 100‐µm cell strainer and add 4 ml of cell isolation medium on top (Fig. 1L).
11 Gently, but with enough force, grind the tissue with the thumb rest of a syringe plunger through the cell strainer, while keeping the cell strainer submerged in cell isolation medium (Fig. 1M‐N). Regularly transfer the filtered cell suspension into the 50‐ml collection tube (Fig. 1O). Add 4 ml of fresh cell isolation medium and continue grinding the tissue until only white connective tissue remains.
12 Discard the remaining connective tissue and rinse the dish with 4 ml of cell isolation medium to recover the remaining cells.
13 Repeat steps 10 to 11 (Fig. 1L‐O) until all tissue is processed (Fig. 1P).
14 Centrifuge cell suspension 5 min at 500 × *g*, room temperature. Remove and discard supernatant (Fig. 1Q).
15 Fill 15 ml Ficoll into a fresh 50‐ml centrifuge tube (Fig. 1R).
16 Resuspend cells in 20 to 40 ml of density centrifugation medium depending on the pellet size to continue with density centrifugation (Fig. 1S) and carefully layer cell suspension onto the Ficoll layer. Tilt the centrifuge tube at a 30° angle to carefully layer the cells by slowly pipetting them onto the Ficoll (Fig. 1T). Carefully tilt back the centrifuge tube; take particular care not to mix the two phases (Fig. 1U).
17 Centrifuge layered cells 30 min at 700 × *g*, room temperature, with acceleration set to 1 and brake set to 0.
18 Transfer centrifuge tube back under the laminar flow hood without disturbing the phases and carefully remove the lymphocyte layer with a 10‐ml serological pipette (Fig. 1V).
19 Collect the isolated cells in a 15‐ml centrifuge tube. If necessary, use 2 or more tubes per donor (Fig. 1W).
20 Centrifuge cell suspension 10 min at 300 × *g*, room temperature. Remove and discard supernatant (Fig. 1X).
21 Resuspend cells in a total of 14 ml of density centrifugation medium. In the meantime, keep the cell suspension on ice.
  If >1 centrifuge tubes were used, merge all cells into 1 centrifuge tube. Be sure to rinse the tubes with additional density centrifugation medium to collect all residual cells, thereby reducing cell loss.

#### Cell counting

22 For counting cells, transfer 10 µl of cell suspension into a 96‐well V‐bottom plate.
  If the cell suspension is quite dense, a 1:10 predilution is recommended. To minimize pipetting errors, a dilution of at least 20 µl cells and 180 µl density centrifugation medium should be made.
23 Pipette 10 µl of trypan blue onto the cells and mix well.
24 Load 10 µl of the mixture into the reusable counting chamber and insert the chamber into the Luna‐FX7 automated cell counter. Adjust focus point and count cells. Generally, cells counted display a diameter of 5 to 30 µm.
  Manual cell counting with a Neubauer chamber or other means of cell counting may be used instead.

#### Cryopreservation of cells

25 Appropriately label cryotubes for identification and better organization between different donors.
26 Centrifuge cells 5 min at 500 × *g*, room temperature. Remove and discard supernatant.
27 Adjust cells to 1 × 10^7^ live cells/ml with cryo medium.
  For optimal cell recovery and high viability after thawing, it is recommended to perform the following steps as fast as possible as soon as the cells come in contact with the freezing medium.
28 Freeze 1 ml aliquot per cryotube by using a repeating pipette to distribute the cell suspension between all cryotubes as quickly as possible.
29 Transfer filled cryotubes into a CoolCell FTS30 freezing container and transfer the container into a –80°C freezer. The next day, move the frozen cells either into a –150°C freezer or liquid nitrogen.
  For short term storage, cells can be stored in –80°C freezers. For storage >6 months, it is recommended to transfer the frozen cells directly to –150°C or liquid nitrogen.
30 The isolated and frozen tonsil/adenoid cells can now be used at any time for Basic Protocols 2 and 3.

## Basic Protocol 2 THAWING OF CRYOPRESERVED TONSIL AND ADENOID CELLS AND ESTABLISHMENT OF IMMUNE ORGANOIDS

This protocol provides a clear framework for thawing human primary immune cells from tonsil and adenoid tissue with the aim of high cell recovery and high cell viability after thawing. Additionally, it gives step‐by‐step instructions on how to set up immune organoids as high‐density single‐cell cultures that provide versatility and scalability in experimental designs.

### Materials

Thawing medium (see recipe)
Frozen human tonsil or adenoid cells from Basic Protocol 1
Bacillol (Hartmann, cat. no. 980214)
Trypan blue solution, 0.4% (Gibco, cat. no. 15250061)
Complete cell culture medium (see recipe)

Water bath
Laminar flow hood
Cryotube racks (Sarstedt, cat. no. 93.856.040)
15‐ml centrifuge tube (Greiner Bio‐One, cat. no. 188271)
1‐ml piston pipette (Mettler Toledo, cat. no. 17014382)
1‐ml pipette tips (Mettler Toledo, cat. no. 30389223)
10‐ml serological pipette (Greiner Bio‐One, cat. no. 607180)
PIPETBOY pipette controller (Integra, cat. no. 155024)
Centrifuge (Eppendorf, cat. no. 5811000015)
Vacuum pump (Integra, cat. no. 158 320)
96‐well V‐bottom micro test plate (Sarstedt, cat. no. 82.1583)
20‐µl piston pipette (Mettler Toledo, cat. no. 17011788)
20‐µl pipette tips (Mettler Toledo, cat. no. 30389225)
Luna‐FX7 reusable cell counter slide (Logos Biosystems, cat. no. L12011)
Luna‐FX7 automated cell counter (Logos Biosystems)
96‐well cell culture plate, suspension, U‐bottom (Sarstedt, cat. no. 83.3925.500)
Incubator (with 5% CO_2_, 37°C)
200‐µl multichannel piston pipette (Mettler Toledo, cat. no. 17013810)
200‐µl pipette tips (Mettler Toledo, cat. no. 30389240)

#### Set up

1 Turn on water bath to 37°C and start the laminar flow hood.
2 Warm thawing medium to 37°C in the water bath.
3 Recover frozen cryotubes containing cells from freezer storage.
  It is important to minimize exposure of the cryotubes to room temperatures (15° to 25°C). If you are not proceeding directly with thawing, place the tubes on dry ice or in a liquid nitrogen container.

#### Thawing cells

4 Wipe the outside of the cryotube containing the cells with Bacillol or 70% ethanol or isopropanol.
5 In a laminar flow hood, twist the cap a quarter‐turn to relieve internal pressure and then retighten.
6 Quickly thaw cells in the 37°C water bath. Remove the vial when a small amount of ice remains. This should take 1 to 2 min.
  Do not vortex the cells!

#### Cell washing and counting

The following steps need to be conducted quickly and efficiently to guarantee high cell viability.

7 Wipe the outside of the vial again with Bacillol, 70% ethanol, or isopropanol.
8 In a laminar flow hood, carefully transfer the cell suspension to a 15‐ml centrifuge tube using a 1‐ml piston pipette and tip.
  At this point, do not resuspend the cells in the cryomedium, just transfer to the other tube.
9 Rinse the vial with 1 ml of thawing medium and add it carefully to the cells in the 15‐ml centrifuge tube without resuspending the cells.
10 Wash cells by adding 12 ml of pre‐warmed thawing medium slowly with a 10‐ml serological pipette and pipette controller into the 15‐ml centrifuge tube. Add it slowly along the wall of the tube.
  Adding medium slowly to the cells reduces osmotic pressure when diluting the cryomedium. This increases the recovery of highly viable cells.
11 Centrifuge the cell suspension 10 min at 300 × *g*, room temperature.
12 Carefully remove the supernatant with a vacuum pump or pipette to ensure the cell pellet is not disturbed.
13 Resuspend the cell pellet by gently pipetting 1 ml of thawing medium into the tube and carefully pipetting up and down.
14 Depending on your cell counter, leave cells in 1 ml of thawing medium or gently add 9 ml of thawing medium with a 10‐ml serological pipette to the centrifuge tube to fill it up to 10 ml for counting.
15 For counting of the cells, transfer 10 µl of cell suspension into a 96‐well V‐bottom plate.
  If the cell suspension is quite dense, a 1:10 predilution is recommended. To minimize pipetting errors, a dilution of at least 20 µl cells and 180 µl density centrifugation medium should be made.
16 Pipette 10 µl of trypan blue onto the cell suspension. Mix well and let cells incubate for ∼30 s with the dye.
17 Load 10 µl of the mixture into the reusable counting chamber and insert the chamber into the Luna‐FX7 automated cell counter. Adjust focus point and count cells. Generally, cells counted display a diameter of 5 to 30 µm.
  Manual cell counting via a Neubauer chamber or other means of cell counting may be used instead.

#### Set up of immune organoids

18 Centrifuge the cell suspension 10 min at 300 × *g*, room temperature.
19 Adjust cells to 2 × 10^6^ cells per 200 µl (i.e., 1 × 10^7^ cells/ml) in complete cell culture medium.
20 Pipette 200 µl of your adjusted cell suspension into each well of a 96‐well U‐bottom cell culture suspension plate.
  Optional: Other cell culture plates can also be used, such as 96‐well flat‐bottom suspension plates or 0.4‐µm polycarbonate trans‐well plates. Cell numbers may need to be adjusted for these different cell culture formats.
21 The immune organoid cultures are now ready for experiments and can be cultured in an incubator at 5% CO_2_ and 37°C for at least 14 days.
  Proper formation of the immune organoids takes ∼16 hr until cells have completely settled to the bottom of the well.
22 Change medium of the immune organoids every other day by carefully removing 50% (100 µl) of the medium with fresh complete cell culture medium using a 200‐µl multichannel piston pipette.
  If the experimental setup leads to stronger cell activation or increased metabolism, medium may have to be changed each day by replacing 50% of the medium with fresh complete cell culture medium.
23 The immune organoids are now ready for analysis, e.g., by flow cytometry (Basic Protocol 3).

## Basic Protocol 3 FLOW CYTOMETRY FOR LONGITUDINAL PHENOTYPICAL PROFILING OF HUMAN TONSIL/ADENOID‐DERIVED IMMUNE ORGANOIDS

The purpose of this protocol is to provide a streamlined framework for the longitudinal analysis of immune organoids using flow cytometry. As an example, we here utilize high‐parametric spectral flow cytometry, but the protocol can also be easily adapted to conventional flow cytometry.

### Materials

Immune organoids in culture (from Basic Protocol 2)
Cell culture medium (see recipe)
1× PBS (Thermo Fisher Scientific, cat. no. 14190169)
Zombie NIR fixable viability kit (BioLegend, cat. no. 423106)
Flow cytometry buffer (see recipe)
Human TruStain FcX (BioLegend, cat. no. 422302)
Normal mouse serum (Invitrogen, cat. no. 10410; alternatively, Merck, cat. no. NS03L)
Antibodies for flow cytometry (see Table 1)
BD Horizon Brilliant Stain Buffer Plus (BD, cat. no. 566385)
eBiosciences FoxP3/Transcription Factor Staining Buffer Set (Thermo Fisher Scientific, cat. no. 00‐5523‐00)
Compensation beads (BioLegend, cat. no. 424602)

Incubator (with 5% CO_2_, 37°C)
96‐well cell culture plate, suspension, U‐bottom (Sarstedt, cat. no. 83.3925.500)
96‐well V‐bottom micro test plate (Sarstedt, cat. no. 82.1583)
Centrifuge (Eppendorf, cat. no. 5811000015)
200‐µl multichannel piston pipette (Mettler Toledo, cat. no. 17013810)
200‐µl pipette tips (Mettler Toledo, cat. no. 30389240)
15‐ml centrifuge tube (Greiner Bio‐One, cat. no. 188271)
1‐ml piston pipette (Mettler Toledo, cat. no. 17014382)
1‐ml pipette tips (Mettler Toledo, cat. no. 30389223)
Eppendorf tubes (Eppendorf, cat. no. 0030121023)
Heat block ThermoMixer C (Eppendorf, cat. no. EP5382000015)
Ice bucket and ice
20‐µl piston pipette (Mettler Toledo, cat. no. 17011788)
20‐µl pipette tips (Mettler Toledo, cat. no. 30389225)
Sony ID7000 spectral analyzer (Sony Biotechnology) or similar
Sony ID7000 analyzer software (Sony Biotechnology) or similar
FlowJo v.10 (Waters)

**Table 1:** Antibodies Used to Identify Immune Cell Populations Within Immune Organoids

Antigen	Fluorochrome	Clone	Company	Cat. no.	RRID	Stock Concentration [µg/ml]	Final dilution
IgD	SparkUV387	IA6‐2	BioLegend	348254	AB_3068144	400	1:400
CD45RA	BUV496	HI100	BD	750258	AB_2874456	200	1:800
CD27	BUV563	M‐T271	BD	741366	AB_2870866	200	1:300
ICOS	BUV737	DX29	BD	749665	AB_2873929	200	1:400
HLA‐DR	BUV805	Tu39	BD	742026	AB_2871322	200	1:600
CCR4	BV421	L291H4	BioLegend	306501	AB_314607	50	1:100
CD38	BV480	HIT2	BD	566137	AB_2739535	300	1:2000
CXCR3	BV605	G025H7	BioLegend	353728	AB_2563157	150	1:100
CD45RO	BV650	UCHL1	BioLegend	304231	AB_2561359	100	1:600
KI‐67	BV750	Ki‐67	BioLegend	350535	AB_2910400	200	1:400
CCR7	BV785	G043H7	BioLegend	353230	AB_2563630	200	1:50
CD4	FITC	RPA‐T4	BioLegend	300506	AB_314074	400	1:600
CCR6	RB670	11A9	BD	771809	AB_3693630	200	1:400
CD19	RB705	SJ25C1	BD	570235	AB_3685597	50	1:800
CD3	RB780	UCHT1	BD	568678	AB_3684461	100	1:400
BCL‐6	PE	K112‐91	BD	561522	AB_10717126	25	1:200
CD25	RY610	M‐A251	BD	758585	AB_3690711	200	1:200
CD21	PE‐Fire640	BU32	BioLegend	354938	AB_3720555	100	1:800
PD‐1	PE‐Fire700	A17188B	BioLegend	621622	AB_2910489	120	1:100
CD127	PE‐Cy7	A019D5	BioLegend	351320	AB_10897098	200	1:200
CXCR5	APC	J252D5	BioLegend	356908	AB_2561817	100	1:50
FOXP3	SparkNIR‐685	206D	BioLegend	320129	AB_2890753	200	1:100
CD8	APC‐Fire810	SK1	BioLegend	344764	AB_2860890	50	1:200

#### Culture of immune organoids

1 Set up immune organoid cultures as described in Basic Protocol 2.
2 Culture cells for the desired length of the experiment in an incubator at 5% CO_2_ and 37°C. Exchange 50% of the used medium with fresh cell culture medium every other day:
  a. Slightly tilt the cell culture plate.
  b. Carefully remove old medium.
  c. Add new medium without disturbing the cell pellet.
3 Analyze cells at the desired analysis timepoints, e.g., day 0, day 1, day 3, and day 10 of culture.
4 Resuspend immune organoids by pipetting thoroughly up and down while avoiding creating bubbles and transfer cells to a 96‐well V‐bottom micro test plate.
  It is important to properly resuspend the immune organoids as some cells tend to stick to the bottom of the wells even though one uses a suspension plate.

#### Surface and intracellular staining of immune organoids for flow cytometry

5 Centrifuge the cell‐containing staining plate 4 min at 400 × *g*, room temperature.
6 Carefully remove supernatant with a 200‐µl multichannel piston pipette by slightly tilting the staining plate, without disturbing the cell pellet.
  Supernatant can also be collected in a sterile collection tube and used for further analyses, e.g., antibody or cytokine production.
7 Resuspend cells in 200 µl of 1× PBS and wash cells by centrifuging 4 min at 400 × *g*, room temperature. Remove and discard supernatant.
8 Resuspend cells again in 200 µl of 1× PBS. From all samples, create a mixed Full Stain control, Unstained control, and Live/Dead control by moving equal parts of each sample to new wells, resulting in a total of 200 µl cell suspension mix for the Full Stain control, and 100 µl each of cell suspension mix for the Unstained control and Live/Dead control.
9 Separate the Live/Dead control 50:50 for a stained and unstained part by pipetting the cells into two Eppendorf tubes (50 µl each). Place the tubes in a heat block at 90°C for 8 min to heat‐kill the cells. Then place the Eppendorf tubes on ice to cool down the cells.
10 Centrifuge the cells 4 min at 400 × *g*, room temperature. Remove and discard supernatant.
11 Stain cells for viability with 100 µl per well of Zombie NIR fixable viability kit at a 1:4000 final dilution in 1× PBS for 15 min at room temperature in the dark. For the Live/Dead control, stain only half of the cells (one Eppendorf tube). Add 100 µl of 1× PBS to the Unstained control and the unstained Live/Dead control.
12 Fill up wells with 100 µl of flow cytometry buffer and centrifuge cells 4 min at 400 × *g*, room temperature. Remove and discard supernatant.
13 Repeat the washing step with 200 µl of flow cytometry buffer and resuspend the two Live/Dead controls (stained and unstained) with 100 µl of flow cytometry buffer each and transfer them back onto your staining plate. Centrifuge cells 4 min at 400 × *g*, room temperature. Remove and discard supernatant.
14 After performing viability staining, block surface Fc Receptors. Create a fresh master mix for surface Fc block by combining Human TruStain FcX (BioLegend, 1:20) and normal mouse serum (Invitrogen, 1:30) in flow cytometry buffer. Add 20 µl of Fc block to each well but skip Unstained control and Live/Dead control; add here 20 µl of flow cytometry buffer. Allow the Fc block to incubate for 15 min at room temperature in the dark.
  Alternative Fc block and mouse serum reagents can also be purchased from other vendors and should be used at pre‐determined concentrations.
15 Directly continue with surface staining.
  a. Prepare a 2× master mix of all surface antibodies.
  b. Pipet the appropriate amount of Brilliant Stain Buffer Plus into an Eppendorf tube: 50% of your total master mix volume should consist of Brilliant Stain Buffer Plus.
  c. Add all antibodies (Table 1) directly into the Brilliant Stain Buffer Plus and make sure to resuspend the mix carefully after each antibody to prevent binding of BUV and BV dyes to one another.
  d. After adding all antibodies, fill up the rest of the volume with flow cytometry buffer.
    For creating your surface master mix, it is also possible to use Brilliant Stain Buffer (also available from other vendors) instead of the Brilliant Stain Buffer Plus. For longitudinal studies, it is recommended to create one big master mix for all days of analyses to minimize variability. In our hands this worked well for the panel provided here. If different fluorophores or antibody combinations are used, it is recommended to perform a test stain to exclude any binding artifacts.
16 Pipette 20 µl of your premade surface antibody master mix to each well. Skip Unstained control and Live/Dead control; instead, add 20 µl of flow cytometry buffer here. Incubate for 1 hr at room temperature in the dark.
17 Fill up wells with 160 µl of flow cytometry buffer and centrifuge cells 4 min at 400 × *g*, room temperature. Remove and discard supernatant.
18 Repeat the washing step with 200 µl of flow cytometry buffer and centrifuge cells 4 min at 400 × *g*, room temperature. Remove and discard supernatant.
19 Fix cells using the eBiosciences FoxP3/Transcription Factor Staining Buffer Set. Use 100 µl per well of the fix/perm solution and incubate cells for 30 min on ice in the dark.
  For achieving higher cell yield, e.g., important for analyses of rare subpopulations within the immune organoids, the dish soap protocol (Burton et al., 2025), which reduces cell loss while still providing decent transcription factor staining (not shown here), can be considered as an alternative. Other commercially available transcription factor staining reagents or kits can also be used at pre‐determined concentrations.
20 Fill the wells with 100 µl of permeabilization buffer from the kit and centrifuge cells 6 min at 600 × *g*, room temperature. Remove and discard supernatant.
21 Repeat the washing step with 200 µl of permeabilization buffer and centrifuge cells 6 min at 600 × *g*, room temperature. Remove and discard supernatant.
22 After fixation and permeabilization, proceed with blocking of intracellular Fc receptors.
  a. Create a fresh master mix by combining Human TruStain FcX (BioLegend, 1:20) and normal mouse serum (Invitrogen, 1:30) in permeabilization buffer.
  b. Add 20 µl of intracellular Fc block to each well. Skip Unstained control and Live/Dead control; instead, add 20 µl of permeabilization buffer here.
  c. Incubate intracellular Fc block for 15 min at room temperature in the dark.
23 Directly proceed with intracellular staining. Prepare a master mix for this step by pipetting the antibodies (Table 1) directly into permeabilization buffer (FoxP3/Transcription Factor Staining Buffer Set). Add 20 µl of your premade intracellular antibody master mix to each well. Skip Unstained control and Live/Dead control; instead, add 20 µl of permeabilization buffer here. Incubate overnight (∼16 hr) at 4°C in the fridge in the dark.
  For learning more about the advantages of overnight staining, refer to Whyte et al. (2022).
24 The next day, fill the wells with 160 µl of permeabilization buffer and centrifuge cells 6 min at 600 × *g*, room temperature. Remove and discard supernatant.
  Be sure to properly resuspend the cells as they tend to aggregate in the bottom of the V‐shaped well.
25 Repeat the washing step with 200 µl of permeabilization buffer and centrifuge cells 6 min at 600 × *g*, room temperature. Remove and discard supernatant.
26 Resuspend cells in 100 µl of flow cytometry buffer.
  If clumps are visible, filtration of the cells may be advisable. For this, a filter mesh of 80‐µm pore size can be used. It should be noted though that filtering will likely result in reduced cell numbers available for downstream analyses.
27 The cells are now ready to be acquired on a flow cytometer, e.g., a Sony ID7000 spectral flow cytometer.
28 For the acquisition of longitudinal data, it is recommend to use fresh single‐stains each day of acquisition. This provides a more robust reference for each fluorophore as there are minor shifts in laser and detector performances each day. For creating the single stains:
  a. Use 20 µl of positive compensation beads and 0.5 µl of each desired fluorochrome.
  b. Incubate the beads for a minimum of 10 min at room temperature in the dark.
  c. Fill up bead mix solution with 60 µl of flow cytometry buffer.
  d. Add your Live/Dead stain to your single stains for the acquisition of the viability single stain for the unmixing.
29 Acquire data on a flow cytometer. If using the Sony ID7000 spectral flow cytometer, manually adjust the unmixing after acquisition in the unmixing adjuster.
30 Export FCS files for further analyses with appropriate software, e.g., FlowJo v10 software.

#### Gating strategy for human tonsil/adenoid‐based immune organoids

31 Begin with gating on all lymphocyte cells (Fig. 2A).

**Figure 2:**
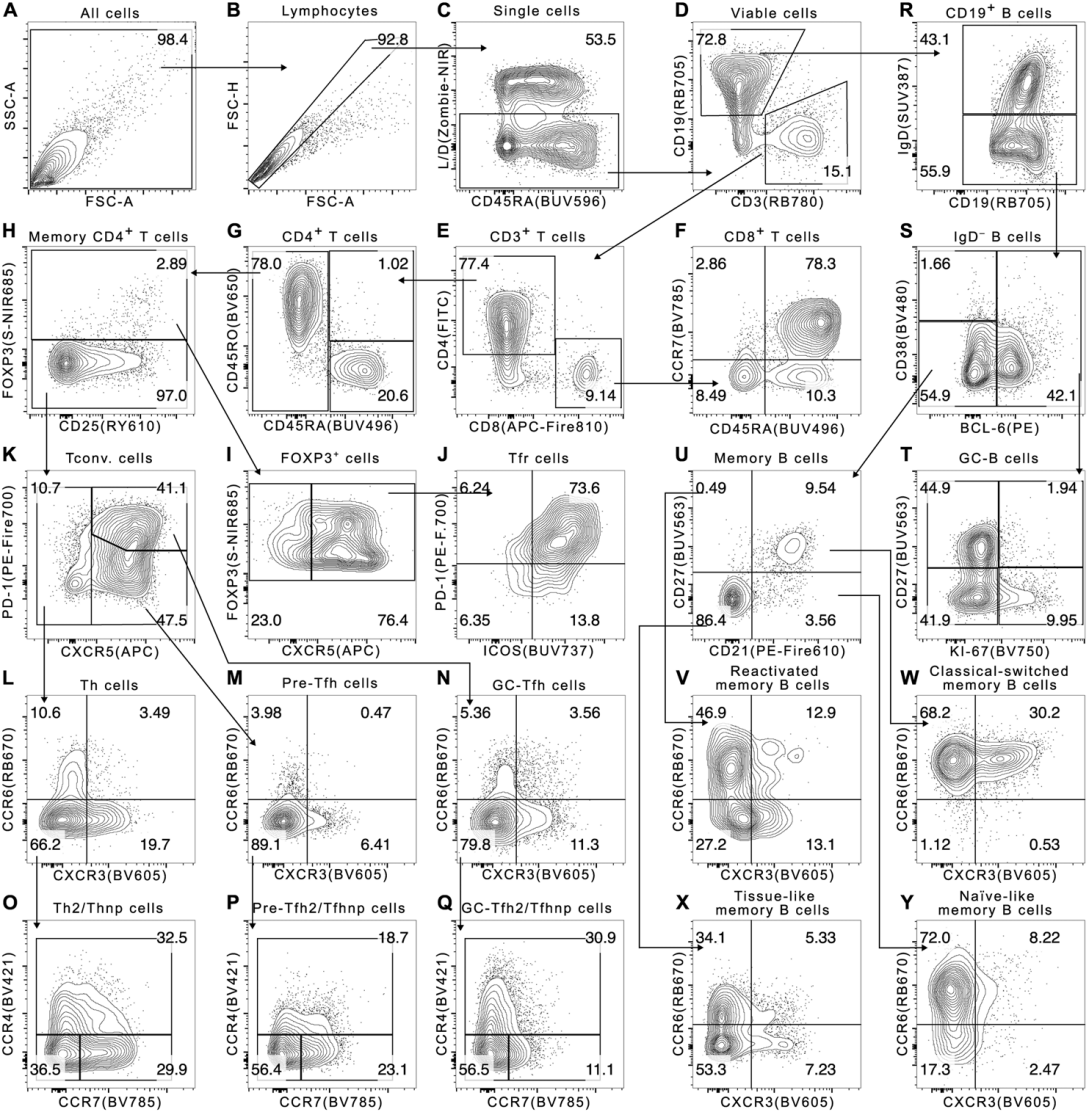
Guided gating strategy for deep phenotyping of human tonsil or adenoid‐derived immune organoids. Representative contour plots of a human tonsil‐derived immune organoid analyzed on day 3 of culture according to Basic Protocol 3.

32 Next, exclude doublet cells by gating FSC‐H vs FSC‐A (Fig. 2B).
33 Dead cells should also be removed from your analyses, e.g., by plotting Zombie‐NIR against CD45RA‐BUV496, as this marker combination provides good separation, and then gating on Zombie‐NIR negative cells (Fig. 2C).
34 Gate B cells and T cells using CD19‐RB705 and CD3‐RB780, respectively (Fig. 2D).
35 Within the CD3^+^ cells, continue gating on CD4^+^ and CD8^+^ T cells (Fig. 2E).
36 Divide the CD8^+^ T cells according to CCR7 and CD45RA expression to differentiate between naïve (CD45RA^+^CCR7^+^), central memory (TCM; CD45RA^–^CCR7^+^), effector memory (TEM; CD45RA^–^CCR7^–^), and effector memory T cells re‐expressing CD45RA (TEMRA; CD45RA^+^CCR7^–^) cell subsets (Fig. 2F).
37 Separate the CD4^+^ T cells according to their activation status using CD45RO and CD45RA markers. It is recommended to gate on memory CD45RA^–^CD45RO^+/–^ cells (Fig. 2G).
  Exclusive expression of CD45RO is not sufficient to define activation in human tonsil immune cells.
38 Memory CD4^+^ T cells are then separated according to their expression of FOXP3 into FOXP3^–^ Tconv cells (Fig. 2H).
39 To further stratify the FOXP3^+^ cells, CXCR5 is used to separate CXCR5^+^ T follicular regulatory (Tfr) cells from CXCR5^–^ Treg cells (Fig. 2I).
40 Using PD‐1 and ICOS expression, Tfr cells can be further separated according to their maturation state into early (PD‐1^–^ICOS^–^), mid (PD‐1^–^ICOS^+^), and late‐GC (PD‐1^+^ICOS^+^) Tfr cells (Fig. 2J) (Ribeiro et al., 2025).
41 Return to the CD4^+^ Tconv cells and continue stratifying these further. Using CXCR5 and PD‐1, separate them into non‐Tfh (= T helper, Th) cells (PD‐1^+/–^CXCR5^–^), pre‐Tfh (PD‐1^low^CXCR5^+^), and GC‐Tfh cells (PD‐1^high^CXCR5^+^) (Fig. 2K).
42 The following gating step can be performed on all Tconv cell subsets (Th, pre‐Tfh, GC‐Tfh cells) to identify polarized cell subsets: CCR6 vs CXCR3 allows for the separation of Th1 (CXCR3^+^CCR6^–^), Th17 (CXCR3^–^CCR6^+^), Th1‐17 (CXCR3^+^CCR6^+^), as well as Th2‐polarized cells together with Thnp (non‐polarized) cells (the remaining CXCR3^–^CCR6^–^ cells) (Fig. 2L‐N).
43 To further separate Th2 and Thnp cells, a second gating step is needed as these two populations are combined within the CXCR3^–^CCR6^–^ double‐negative gate. Use CCR4 and CCR7 to identify Th2 cells (all CCR4^+^ cells). Divide CCR4^–^ Thnp cells into CCR4^–^CCR7^+^ (Thnp CCR7^+^) or CCR4^–^CCR7^–^ (Thnp CCR7^–^) cells (Fig. 2O‐Q).
44 After defining the main CD8^+^ (naïve, TCM, TEM, TEMRA cells) and CD4^+^ T cells subsets (Treg, Tfr, Th, pre‐Tfh, GC‐Tfh cells) and their polarized counterparts, activation markers can be quantified depending on their biological relevance for the specific subset, e.g., CD27, ICOS, HLA‐DR, CCR4, CD38, CXCR3, CCR7, CCR6, CD25, PD‐1, and CD127 (Fig. 3).

**Figure 3:**
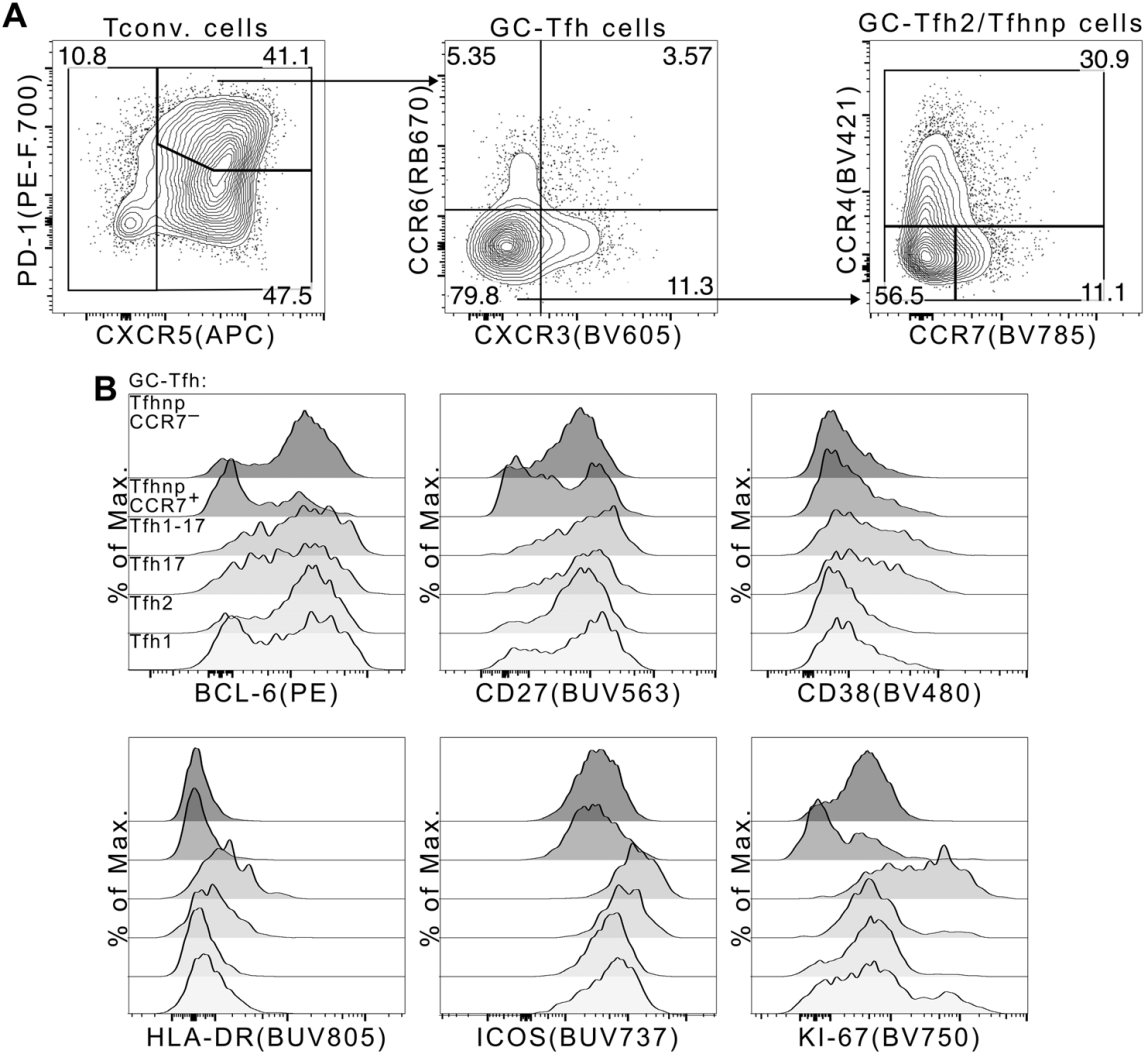
Activation marker expression by GC‐Tfh cell subsets. (**A**) Representative gating strategy to define GC‐Tfh cell sub‐populations in a representative human tonsil‐based immune organoid analyzed on day 3 of culture according to Basic Protocol 3. (**B**) Histograms show expression of various activation makers by the indicated GC‐Tfh cell sub‐populations.

45 Return to the CD19^+^ B‐cell gate (Fig. 2D) and separate the B cells according to their activation state by using IgD expression. Continue with IgD^low/–^ B cells (Fig. 2R).
46 Using CD38 and BCL‐6, define the IgD^low/–^ B cells being either plasmablasts (CD38^hi^BCL‐6^–^), memory B cells (CD38^low/–^BCL‐6^–^), or GC‐B cells (CD38^–/+^BCL‐6^+^) (Fig. 2S).
47 Continue by further stratifying GC‐B cells according to CD27 and Ki‐67 expression. This allows for the distinction of centrocytes (CD27^low/–^Ki‐67^–^), centroblasts (CD27^low/–^Ki‐67^+^), post‐GC early memory B cells (CD27^high^Ki‐67^–^), and early plasmablasts (CD27^high^Ki‐67^+^) (Fig. 2T).
48 Return to the memory B‐cell gate (Fig. 2S) and gate on these cells to separate them further into classical switched memory B cells (CD27^+^CD21^+^), naïve‐like memory B cells (CD27^–^CD21^+^), tissue‐like memory B cells (CD27^–^CD21^–^), and reactivated memory B cells (CD27^+^CD21^–^) (Fig. 2U).
49 Finally, CCR6 and CXCR3 can be used to further distinguish the functional phenotype of these four memory B‐cell subsets.
50 All gated cell populations can now be assessed and quantified for the underlying research question.

## REAGENTS AND SOLUTIONS

### Cell isolation medium

500 ml RPMI 1640 Medium, GlutaMAX Supplement (Thermo Fisher Scientific, cat. no. 61‐870‐044)
5 ml of 100× MEM sodium pyruvate (1× final; Thermo Fisher Scientific, cat. no. 11360039)
5 ml of 100× MEM nonessential amino acids (1× final; Fisher Scientific, cat. no. 11140035)
5 ml of 100× penicillin‐streptomycin (10,000 U/ml) (1× final; Thermo Fisher Scientific, cat. no. 15140122)
5 ml of 250 µg/ml amphotericin B (2.5 µg/ml final; Thermo Fisher Scientific, cat. no. 15290026)
75 ml fetal bovine serum (FBS), heat inactivated (15% v/v final; Thermo Fisher Scientific, cat. no. A5256801)
Invert well
Store up to 1 month at 4°C in the dark

### Complete cell culture medium

100 ml cell isolation medium (see recipe)
1 ml of 100× insulin‐transferrin‐selenium (ITS ‐G) (1× final; Biomol, cat. no. PB180429.10)
1 ml of 50 mM 2‐mercaptoethanol (50 µM final; Thermo Fisher Scientific, cat. no. 31350010)
1 ml of 1 M HEPES (10 mM final; Thermo Fisher Scientific, cat. no. 15630056)
Store up to 1 month at 4°C in the dark

### Cryo medium

45 ml FBS, heat inactivated (90% v/v final; Thermo Fisher Scientific, cat. no. A5256801)
5 ml dimethyl sulfoxide (DMSO), BioUltra, for molecular biology, ≥99.5% (GC) (5% v/v final; Sigma‐Aldrich, cat. no. 41639‐100ML)
Invert well
Store up to 1 month at 4°C in the dark

### Density centrifugation medium

500 ml Dulbecco's phosphate‐buffered saline (DPBS), no calcium, no magnesium (Thermo Fisher Scientific, cat. no. 14190169)
10 ml FBS, heat inactivated (2% v/v final; Thermo Fisher Scientific, cat. no. A5256801)
Invert well
Store up to 1 month at 4°C in the dark

### Flow cytometry buffer

1 L H_2_O, Millipore
2 tablets of PBS (1× final; Thermo Fisher Scientific, cat. no. 18912‐014)
0.5% bovine serum albumin (BSA) (Sigma‐Aldrich, cat. no. A2153‐100G)
2 mM EDTA (VWR, cat. no. E177‐100ML)
NaN_3_ dissolved in PBS (0.01% final; Carl Roth, cat. no. 4221.1)
Store up to 6 months at 4°C in the dark

### Thawing medium

500 ml RPMI 1640 Medium, GlutaMAX Supplement (Thermo Fisher Scientific, cat. no. 61‐870‐044)
50 ml FBS, heat inactivated (2% v/v final; Thermo Fisher Scientific, cat. no. A5256801)
Store up to 1 month at 4°C

## COMMENTARY

### Critical Parameters

#### Basic Protocol 1

In order to produce robust results from immune organoids, it is critical to process the tonsil and adenoid tissue within a short time frame, i.e., limiting the time of tissue processing to a minimum. Limit the decontamination step to overnight with a maximum of 24 hr. Perfusion of the tissue is not guaranteed when just placed into cell culture medium. A further critical step is the grinding of the tissue through a 100‐µm cell strainer. If too much force is used, cells die during processing and this will later result in reduced viability during immune organoid culture. Special care should be taken when freezing the isolated cells. Prolonged contact of cells with freezing medium at room temperature leads to increased cell death. When freezing the isolated cells, work fast and use chilled freezing medium directly from the fridge (4°C). For long‐term storage (>6 months), it is highly recommended to transfer the frozen cells after overnight freezing into a –150°C freezer or liquid nitrogen.

#### Basic Protocol 2

When thawing the isolated tonsil/adenoid cells, there are several critical steps that need to be carried out to obtain robust results. All steps of the thawing procedure should be executed as quick and efficient as possible. Reduce the exposure of the frozen cells to room temperature to a minimum. Use dry ice if you need to store cryotubes short‐term until the actual thawing process is started. When adding the thawing medium to the cells (step 10), do so very slowly along the tube walls to the cells or drop‐by‐drop. This will reduce the osmotic pressure caused by the dilution of the cryomedium, which will increase cell recovery and viability of thawed cells. Thawed cells displaying a viability of ≤80% will result in immune organoids with poor viability.

#### Basic Protocol 3

When performing longitudinal flow cytometry analyses, staining mixes should be prepared as one master mix for all time points to reduce variability in the staining result. It is important to test the master mix beforehand to make sure that the antibody combination used is not binding to each other and no artifacts are generated. In our hands, the surface antibody mix used in this protocol stays stable for up to 10 days for spectral flow cytometry analyses. We did not test this further but using it for up to 14 days should be possible. For the preparation of the surface stain, it is critical to pipet all antibodies directly into the Brilliant Stain Buffer Plus; this prevents binding of BUV or BV dyes to each other. If several PE‐tandem dyes are used, consider adding tandem stabilizer (BioLegend, cat. no. 421802) to your master mix. The live/dead stain must always be prepared fresh right before staining as the amine dyes are susceptible to being hydrolyzed by water. For intracellular staining, testing of the stability of the antibodies in permeabilization buffer should be carried out, as the chemicals in the permeabilization buffer may affect the fluorophores. For this staining protocol, we prepared them fresh for each day of analysis.

### Troubleshooting

See Table 2 for a troubleshooting guide for the tonsil/adenoid‐derived immune organoid setup and flow cytometry protocol.

**Table 2:** Troubleshooting Guide for Tonsil/Adenoid‐derived Immune Organoid Setup and Flow Cytometry Protocol

Problem	Possible cause	Solution
Bacterial contamination in organoid culture	No proper decontamination of the crypts of the tonsils; no addition of 10× penicillin‐streptomycin for bigger tissue pieces	Longer decontamination of tissue; addition of more penicillin‐streptomycin (final maximum: 10×)
Poor viability after tissue processing	Excessive grinding with too much force on the tissue using the cell strainer	Reduce force and grind more carefully
Poor viability after thawing of frozen cells	Immersing the cells too quickly in thawing medium; poor donor‐specific viability	Add thawing medium drop‐by‐drop; use different donor(s) as some donors show overall poor viability after thawing
No live/dead signal on stained cells	Used >1‐day‐old master mix of the Zombie‐NIR dye	Always prepare a fresh live/dead stain at day of staining
Biologically unlikely double‐positive artefacts on the stained cells	Binding of BUV or BV dyes to each other causing double‐positive artefacts	Pipet all antibodies directly into Brilliant Stain Buffer Plus and properly resuspend after each antibody; change one of the problem‐causing fluorophores

### Understanding Results

The primary objective of this protocol is to provide a guided protocol for the isolation of human tonsil/adenoid‐derived immune cells and their set‐up as immune organoid cultures, as well as a detailed framework for analyzing the immune organoids by flow cytometry. Figure 1 provides the reader with a detailed step‐by‐step guide, illustrating the processing of the tonsil/adenoid tissue for experimental use. Utilization of frozen samples allows for more flexibility in assay design and enables the experimenter to thaw multiple donors at once, thereby accommodating for donor variability and reducing batch effects between different experimental runs. Figure 2 presents a detailed gating strategy to identify the main T and B cell subsets present in immune organoids, which allows for in‐depth analyses of human immune responses in SLO‐like environments. The provided gating strategy allows CD4^+^ T cells to be stratified into naïve and memory cells (Fig. 2G). The memory cells can be further separated into conventional and regulatory T cells (Fig. 2H). Regulatory T cells can be further divided to Treg, pre‐Tfr, and Tfr cells (Fig. 2I). Tfr cells can be separated into early‐, mid‐, and late‐GC state (Fig. 2J). T conventional cells are further separated by the expression of PD‐1 and CXCR5, allowing for the identification of Th, pre‐Tfh, and GC‐Tfh cells (Fig. 2K). These three main subsets can be then separated into their polarizations, including Th1, Th2, Th17, Th1‐17, and Thnp (Fig. 2L‐Q). CD8^+^ T cell subsets include Tnaïve, TCM, TEM, and TEMRA (Fig. 2F). B cells can be analyzed in‐depth as well, identifying IgD‐negative B cells (Fig. 2R), and separating these further into plasma cells, GC‐B cells, and memory B cells (Fig. 2S). GC‐B cells can be further separated into centrocytes, centroblasts, early memory B cells, and early plasmablasts (Fig. 2T). To further distinguish memory B cells (MBCs), CD27 and CD21 are used to separate these cells into classical switched MBCs, naïve‐like MBCs, tissue‐like MBCs, and reactivated MBCs (Fig. 2U). Lastly, these MBC subsets can be gated according to their functional phenotype into dual‐polarized MBCs, anti‐viral primed MBCs, classical MBCs, and mucosal associated MBCs (Fig. 2V‐Y). We further included multiple activation markers relevant to T cells into this panel to assess the activation state and phenotype of the identified T cell subsets, e.g., BCL‐6, CD27, CD38, HLA‐DR, ICOS, and KI‐67 for GC‐Tfh cell subpopulations (Fig. 3). In summary, this staining panel provides deep phenotyping of T and B cell subpopulations relevant to studying adaptive immunity in tonsil/adenoid‐derived immune organoids.

To illustrate the dynamics of adaptive immune cell populations within the immune organoid model, 5 tonsil‐derived and 5 adenoid‐derived organoids were cultured for 10 days as described in Basic Protocol 2. Cultures were sampled at days 0, 1, 3, and 10 and analyzed by spectral flow cytometry as outlined in Basic Protocol 3. Tonsil‐derived immune organoids showed slightly lower viability than adenoid‐derived immune organoids (Fig. 4). The frequencies of the major adaptive immune cell subsets, CD4^+^ T cells, CD8^+^ T cells, and CD19^+^ B cells, did not differ much between the two immune organoid types during the time frame of the experiment (Fig. 4). CD4^+^ T cell frequencies increased over the 10‐day period of culture, whereas CD8^+^ T cell frequencies declined modestly by day 10 (Fig. 4). CD19⁺ B cell frequencies decreased through day 3 and remained stable thereafter (Fig. 4).

**Figure 4:**
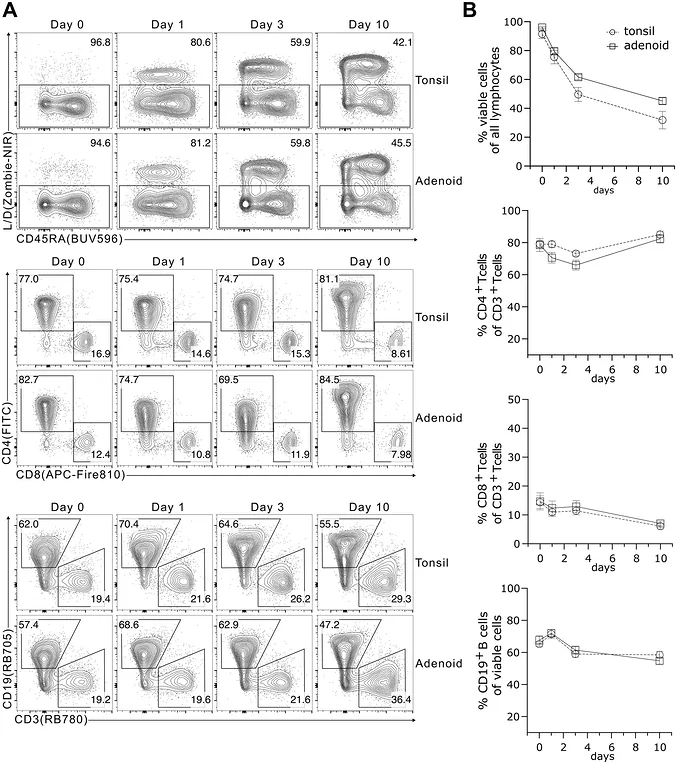
Dynamics of major immune cell subsets in human immune organoids. (**A**) Tonsil and adenoid‐based immune organoids were cultured for 10 days as described in Basic Protocol 2 and then analyzed by spectral flow cytometry on day 0, 1, 3, and 10 according to Basic Protocol 3. Representative flow cytometry contour plots display the frequency of viable cells, CD19^+^ B cells, CD4^+^ T cells, and CD8^+^ T cells. (**B**) Quantification of the data in (**A**). The mean values of biological replicates were calculated and are indicated by the symbols. Error bars indicate *SEM*. Each symbol type represents 5 donors (*n* = 5 donors per tonsil‐derived and *n* = 5 per adenoid‐derived immune organoids). Donors where all age 3 to 4 and gender was equally distributed.

### Time Considerations

#### Basic Protocol 1

Processing and freezing time required for 1 donor is 2 to 3 hr. We recommend limiting tissue processing to a maximum of 2 donors per researcher at a time.

#### Basic Protocol 2

Time requirements for thawing and culture setup depend on the number of donor samples to be included. For 10 different donors and using an automated cell counter, plan for ∼1.5 hr from start to complete culture setup. Time required for cell counting represents a bottleneck in this workflow.

#### Basic Protocol 3

For the first day of staining of the immune organoids for flow cytometry, plan for ∼3 hr from start until the intracellular staining mix is added to the cells, followed by overnight incubation at 4°C. The following day, allow for an additional 15 min for the remaining staining steps and 20 min for setting up single stains, which can be performed in parallel to the final intracellular staining steps.

### Statistical and Data Analysis

Flow cytometry data were analyzed with FlowJo 10.10.1 (FlowJo LLC). Data presented here are shown as mean ± *SEM*. Figures were created in Prism 11.0.2 (GraphPad) or Inkscape 1.4.3 (Inkscape).

### Author Contributions

**Maximilian Moll**: Conceptualization; formal analysis; investigation; methodology; writing—original draft; writing—review and editing. **Klaus Eichhorn**: Resources; writing—review and editing. **Thorsten Send**: Resources; writing—review and editing. **Dirk Baumjohann**: Conceptualization; formal analysis; funding acquisition; investigation; methodology; project administration; supervision; writing—original draft; writing—review and editing.

### Conflict of Interest

The authors declare no competing interests.

## Data Availability

The data that support the protocol are available from the corresponding author upon reasonable request.
